# 3D segmentations of neuronal nuclei from confocal microscope image stacks

**DOI:** 10.3389/fnana.2013.00049

**Published:** 2013-12-27

**Authors:** Antonio LaTorre, Lidia Alonso-Nanclares, Santiago Muelas, José-María Peña, Javier DeFelipe

**Affiliations:** ^1^Department of Functional and Systems Neurobiology, Instituto Cajal (Consejo Superior de Investigaciones Científicas)Madrid, Spain; ^2^Laboratorio Cajal de Circuitos Corticales, Centro de Tecnología Biomédica, Universidad Politécnica de MadridMadrid, Spain; ^3^DATSI, Facultad de Informática, Universidad Politécnica de MadridMadrid, Spain

**Keywords:** 3D reconstruction, automatic segmentation, cerebral cortex, image processing, neuron

## Abstract

In this paper, we present an algorithm to create 3D segmentations of neuronal cells from stacks of previously segmented 2D images. The idea behind this proposal is to provide a general method to reconstruct 3D structures from 2D stacks, regardless of how these 2D stacks have been obtained. The algorithm not only reuses the information obtained in the 2D segmentation, but also attempts to correct some typical mistakes made by the 2D segmentation algorithms (for example, under segmentation of tightly-coupled clusters of cells). We have tested our algorithm in a real scenario—the segmentation of the neuronal nuclei in different layers of the rat cerebral cortex. Several representative images from different layers of the cerebral cortex have been considered and several 2D segmentation algorithms have been compared. Furthermore, the algorithm has also been compared with the traditional 3D Watershed algorithm and the results obtained here show better performance in terms of correctly identified neuronal nuclei.

## 1. Introduction

The development of methods that accurately estimate the number of cells (neurons and glia) in the brain is a major challenge in neuroscience. Numerous methods have been developed to estimate the number of cells in a given volume of brain tissue [e.g., stereology Sterio (1984); Williams and Rakic (1988); West and Gundersen (1990)]. However, there are often discrepancies in the results from different laboratories due to the diverse methodologies and mathematical approaches used to obtain the estimates (Beaulieu, 1993; Keller and Carlson, 1999; Herculano-Houzel and Lent, 2005). Moreover, most of the methods for neuron counting are based on manual detection and are time consuming and user-dependent. The automated segmentation of neurons would be a more efficient and unbiased alternative. Therefore, the development of efficient and automatic methods to determine the actual number of cells is a major aim in neuroanatomy.

Automatic techniques have attempted to estimate the number of neurons via various different two-dimensional (2D) automated algorithms, such as threshold-based (Wu et al., 2000), Watershed (Lin et al., 2003) and model-based [reviewed in Oberlaender et al. (2009)]. Moreover, automated three-dimensional (3D) approaches have been developed to generate a landmark set that represents the neuronal somata within an image stack (Oberlaender et al., 2009).

However, to the best of our knowledge, the techniques that are currently available for 3D segmentation do not solve problems such as over-segmentation in Watershed techniques [see Oberlaender et al. (2009) and the references therein] and do not report important data such as the size and shape of the cells [e.g., Bai et al. (2009); Oberlaender et al. (2009)]. Furthermore, most of these methods do not work well in brain regions with a high density of cells (since adjacent cells are difficult to discriminate). The selective labeling of neurons in brain sections represents an additional problem. The most common method that attempts to do this is immunohistochemistry using anti-NeuN antibodies. NeuN reacts with most neuronal cell types throughout the brain but the immunostaining is localized not only in the nucleus (round shape), but also in the cytoplasm of the neurons. This cytoplasmic staining confers to the labeled neurons an irregular shape, making it very difficult to segment the images obtained with this method. Alternatively, DAPI is a fluorescent stain that is used extensively in fluorescence microscopy to label the nuclei of cells, and the images obtained with this labeling are much easier to segment. However, DAPI labels both neurons and glia.

For these reasons we have developed a method based on a new accurate technique for 2D cells segmentation (LaTorre et al., 2013). Our method allows cells to be automatically segmented in 3D and provides accurate data concerning their spatial distribution, size and shape. In order to resolve the problem of the irregular shape of NeuN labeled neurons, we combined the two methods of staining (NeuN and DAPI) to discard non-neuronal nuclei (removing those elements in the DAPI channel not present in the NeuN images), thereby allowing the selective analysis of only the nuclei from neurons or glial cells (LaTorre et al., 2013).

We have focused on counting neuronal cells located in the rat neocortex, which is a multi-laminated and highly organized structure with different cell densities in different layers, making it ideal for testing the reliability of the method in different conditions. In the present study, we demonstrate that it is possible to obtain an accurate number of neurons in any layer of the neocortex using our method.

## 2. Materials and methods

### 2.1. Tissue preparation

Male Wistar rats sacrificed on postnatal day 14 were used for this study. Animals were administered a lethal intraperitoneal injection of sodium pentobarbital (40 mg/kg) and were intracardially perfused with 4 paraformaldehyde in 0.1 M phosphate buffer. The brain was then extracted from the skull, fixed and sliced into coronal sections (50 μm) that were collected serially. All animals were handled in accordance with the guidelines for animal research set out in the European Community Directive 2010/63/EU, and all procedures were approved by the local ethics committee of the Spanish National Research Council (CSIC). Sections containing the hindlimb region of the somatosensory cortex [S1HL; by Paxinos and Watson (2007)] were stained using immunofluorescence staining. Free floating sections were incubated for 2 h in blocking solution: phosphate buffer (PB: 0.1 M, pH 7.4) with 0.25 Triton-X and 3 normal horse serum (Vector Laboratories, Burlingame, CA, USA). The sections were then incubated overnight at 4°C with a mouse anti-neuron-specific nuclear protein (NeuN, 1: 2000, Chemicon, Temecula, CA, USA). After rinsing in PB, the sections were incubated for 1 h at room temperature with an Alexa Fluor 488 goat anti mouse (1:1000, in blocking solution; Molecular Probes). Thereafter, the sections were stained with a solution containing 105 mol/L of the fluorescent dye 4, 6-diamidino-2-phenylindole (DAPI; Sigma D9542, St Louis, USA). After staining, the sections were mounted with ProLong Gold Antifade Reagent (Invitrogen).

### 2.2. Image acquisition

**Figure 1:**
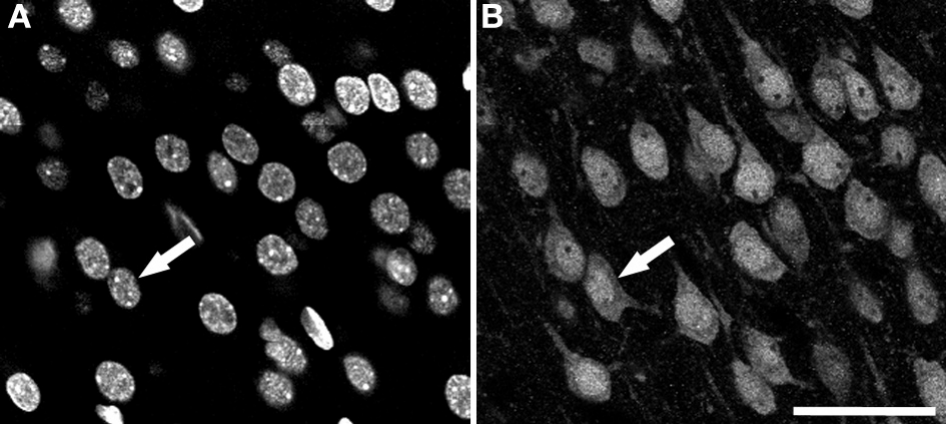
**Examples of confocal images from the rat cerebral cortex**. These two images were taken from the same field and plane of a section double-labeled for DAPI and NeuN. **(A)** DAPI channel image showing nuclei from all cell types. **(B)** NeuN channel image showing the nuclei, the soma and proximal processes of immunoreactive neurons. Arrows indicate the same neuron in both channels. Scale bar in **(B)**: 60 μm.

Sections were examined with a Zeiss 710 confocal laser scanning system (Carl Zeiss Microscopy, Germany). NeuN-immunoreactivity (-ir; for neurons) and DAPI staining (for nuclei of all cell types) fluorescence was recorded through separate channels. Confocal image stacks of 40-50 planes were obtained with an EC PL NEO 40x immersion lens (N.A. 1.3), using a z-step of 1 μm and a scanning resolution of 512x512 pixels (pixel size 0.5 μm). An example of both types of images is shown in Figure 1.

### 2.3. 3D reconstruction algorithm

#### 2.3.1. Overview

The 3D reconstruction algorithm proposed in this paper works with the output of a 2D segmentation algorithm. This 2D algorithm can be of any kind, as will be discussed in the following section, and may be adjusted to meet the special needs of a particular segmentation problem (i.e., depending on the characteristics of the images in question). The rationale behind this approach is that, by separating 3D reconstruction from 2D segmentation, it is possible to generalize an algorithm that works on a wide range of tissue samples, acquisition methods or even application fields. Therefore, the 3D algorithm deals only with 2D-segmented regions of a sequence of continuous stacks. This integrative approach also opens up the possibility of applying this algorithm to new 2D segmentation methods as common refinement phase and also allows 3D segmented elements to be produced.

The workflow of the proposed algorithm is as follows. The original images obtained from the confocal microscope are processed by a 2D segmentation algorithm that must separate foreground objects from the background; remove non-neuronal cells and artifacts from the image; and attempt to split existing clumps of cells to identify individual cells. The output of this 2D segmentation algorithm is a stack of individually labeled 2D regions of candidate cells. The 3D reconstruction algorithm must then find a suitable reconstruction of 3D cells from adjacent 2D cells in different slices, as will be described in detail in the following sections.

#### 2.3.2. 2D segmentation algorithms

For our experiments, we have used a 2D segmentation algorithm that is divided into two main phases: initial segmentation and division of clusters. Firstly, the original images are processed to separate foreground objects from the background. Secondly, each identified foreground object is analyzed to attempt to determine if that object is actually a cluster of touching/overlapping cells. In the case that an object is identified as a cluster of cells, it is divided into multiple individual cells.

In order to provide more insight into the factors that influence the performance of the proposed 3D reconstruction algorithm, we have used two different algorithms for each of the phases of the 2D segmentation algorithm. For the initial segmentation phase, we considered a Two-steps Binarization algorithm (LaTorre et al., 2013), which is tailored to this particular type of images, and more general state-of-the-art algorithms such as Level Set Methods (Li et al., 2010). For the second phase (division of clusters of cells), we have used a variation of the Clump Splitting Algorithm (LaTorre et al., 2013) and the well-known Watershed algorithm (Beucher and Lantuéjoul, 1979). The combination of these four algorithms leads to the following four configurations:

- Two-step Binarization + Clump Splitting (BinCS),
- Two-step Binarization + Watershed (BinWS),
- Level Set Methods + Clump Splitting (LSCS) and
- Level Set Methods + Watershed (LSWS).

Each of these four configurations follow the same aforementioned two-phase scheme: initial segmentation and division of clusters of cells. These techniques have been previously used in the context of 2D cell identification and, in particular, in the segmentation of neuronal cell nuclei, which is the application that is dealt with in the present study.

Since Level Set Methods and Watershed are well-known algorithms, we will limit ourselves to a brief description of the other two considered algorithms.

**Figure 2:**
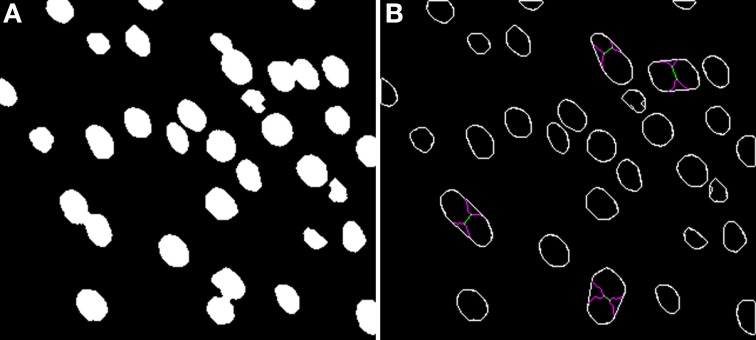
**Binary image (A) computed from the DAPI Channel and its associated segmented image (B)**. Concavities that are considered by the Clump Splitting algorithm are shown in purple, whereas division lines for cells are shown in green and orange, depending on the type of division carried out [details on this issue can be found in LaTorre et al. (2013)].

The Two-step Binarization algorithm takes the original images (like those shown in Figure 1) and separates foreground objects from background, creating a binary image. The idea behind this two-step binarization is, first, to try to obtain a rough estimate of the binary image by means of a global thresholding algorithm and, second, to refine this estimate by using only local information. This must be done in order to better deal with the intensity variations in different regions of the images and the noise that is inherent to this type of image. Additionally, several morphological transformations and filters are used at different points to improve the quality of the overall binarization process. The binarized images coming from both channels (DAPI and NeuN) are combined in order to discard objects that are not neurons (objects in the DAPI channel not present in the NeuN channel). This is necessary for two reasons: (1) it is always better to be able to differentiate different types of cells since, in this way, we will be able to segment these other cells in the event that we are interested in them and; (2) neurons' nuclei (DAPI channel) are round-shaped, whereas neurons (NeuN channel) have different shapes, which makes it easier to work with the DAPI channel. An example of the resulting images coming from this first phase is presented in Figure 2A. The result of using the Level Set Methods in our experimentation yields similar (binary) images, although of different quality, as will be discussed later.

Turning to the Clump Splitting algorithm (and the corresponding Watershed algorithm), these algorithms take the binary images coming from the previous phase and split those groups of cells that are tightly-coupled in the original images and have been binarized together. A full description of the two-step binarization algorithm can be found in LaTorre et al. (2013). An example of the segmented images obtained at the end of the whole process is shown in Figure 2B.

#### 2.3.3. 3D reconstruction algorithm

**Figure 3:**
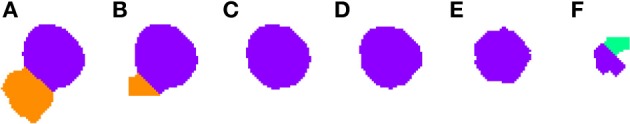
**Segmented nuclei prior to the first phase of the 3D reconstruction**. A neuronal nucleus that has been properly segmented in five slices **(A–E)** by the 2D algorithm has been over-segmented in the last one **(F)** into two parts (labeled in green and purple).

**Figure 4:**
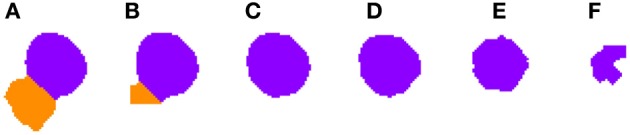
**Segmented nuclei after over-segmentation correction by joining most overlapping blocks**. Note the differences in **(F)** slice between Figure 3 and this figure. The incorrectly segmented neuronal nucleus in 2D (Figure 3F) has been corrected and the division has been removed, whereas the other five slices **(A–E)** have been segmented as in Figure 3.

**Figure 5:**
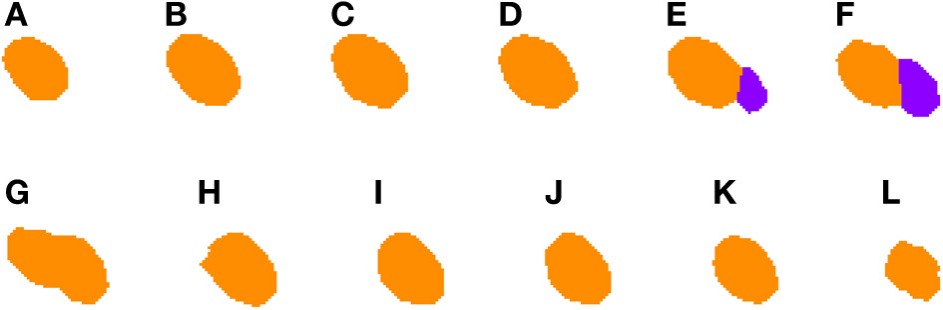
**Segmented nuclei prior to under-segmentation correction**. This figure shows two adjacent neuronal nuclei. First nucleus appears in slices **(A–G)**, whereas the second one appears in slices **(E–L)**. Both nuclei overlap in slices **(E–G)** and the 2D algorithm fails to split them in slice **(G)**, making the 3D reconstruction algorithm unable to segment them properly.

**Figure 6:**
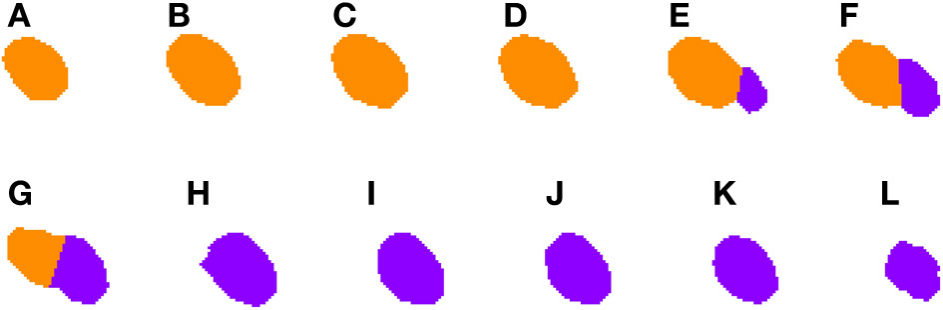
**Segmented nuclei after under-segmentation correction which splits blocks that overlap to a large extent with previous blocks**. The same two neuronal nuclei incorrectly split in Figure 5 are now correctly divided. The correction step divides the nuclei in the overlapping slices **(E–G)** and assigns correct labels to each of them: orange to the first nucleus (slices **A–G**) and purple to the second one (slices **E–L**).

**Figure 7:**
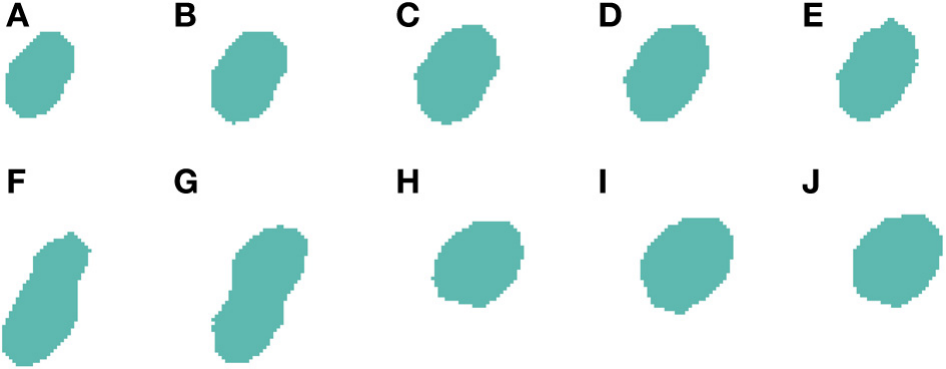
**Segmented nuclei prior to under-segmentation correction by examining centroids**. The 2D algorithm is unable to correctly segment the two neuronal nuclei [one in slices **(A–G)** and another one in slices **(F–J)**] that touch in **(F,G)**, which results in the 3D reconstruction algorithm incorrectly considering that there is a single nucleus in this segmentation.

**Figure 8:**
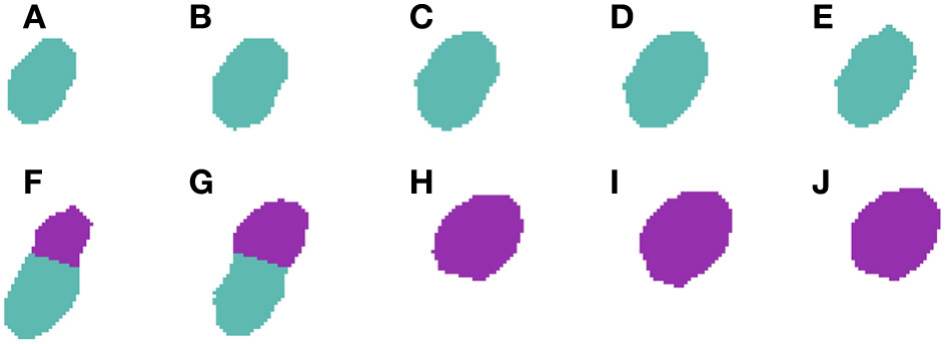
**Segmented nuclei after under-segmentation correction by examining centroids**. This improvement is able to detect a large displacement of the nucleus centroid in **(F,G)** and thus divide both nuclei at these slices, resulting in one nucleus in slices **(A–G)** and another one in slices **(F–J)**.

**Figure 9:**
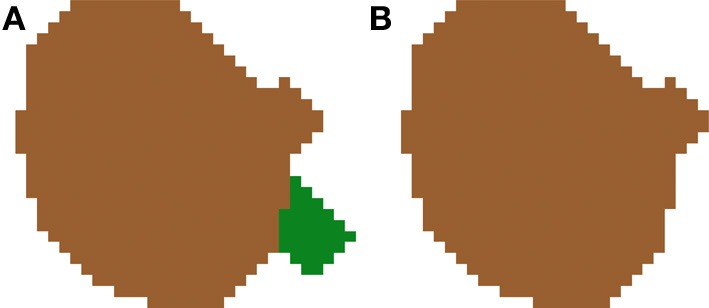
**Segmented nuclei before (A) and after (B) removing small artifacts introduced by the 2D segmentation**. Most errors of this type are typically produced when dye staining also fills portions of the closest part to the nucleus, causing an irregular shaped cell body appearing in a single slice. Due to this shape, these regions are sometimes labeled as different 2D structures in one or two slices. In our approach, these small structures are removed from the final segmentation.

In this section of the paper, Figures 3–9 illustrate the application of each of the main steps of the algorithm to a sample input image. This sample input image is different for each step since it was necessary to choose an image that was relevant to the particular issue addressed by the (algorithm) step in question.

The algorithm starts with a clump of cells—a 3D structure of cells that are already segmented in 2D (as shown in Figure 2B). This stack is first subject to a flood-fill algorithm in which every independent 3D connected component is labeled (“connected component” here refers to components with adjacent foreground pixels). For convenience, the line that splits two cells is randomly assigned to one of the cells involved in the division. Once every 3D connected component has been identified, each of them will be processed individually. For this purpose, their 3D bounding boxes are computed, as will be seen in the figures shown in the remainder of this section.

#### 2.3.4. 3D segmentation of connected components

The first phase of the algorithm involves the analysis of the aforementioned 3D connected components. These components may correspond to a single cell nucleus (the most common case) or to a group of touching/overlapping cell nuclei that were already split in 2D. The objective of this first phase is to obtain 3D reconstructions of individual cell nuclei by processing the information of the 2D segmentation from the first to the last slice, combining 2D cell nuclei that overlap to a large extent. Furthermore, this process is able to correct some common errors in 2D segmentation, such as over- or under-segmentation, as will be seen in the following examples. Algorithm 1 describes how this 3D reconstruction is carried out. In this algorithm, we have used the following notation: *S*_*i*_ represents each of the slices of the stack of images; *C*_*i*_ names each of the 3D cells, whereas *c*_*i*,*j*_ represents a 2D slice of a cell, where *i* is the slice number and *j* is the 3D cell that this 2D cell belongs to; finally, δ_1_ and δ_2_ are two control parameters used by the algorithm to decide if two structures overlap sufficiently.

**Algorithm 1:** **3D Reconstruction Algorithm**.

1:	**for** every 3D connected component **do**
2:	Obtain the 3D Bounding Box of the connected component.
3:	Initialize a set of cells with the 2D information of the first slice (*S*_0_). Slices are numbered in increasing order and they are processed from top to bottom. If this first slice contains *k* 2D cells, each of the 3D cells will be numbered accordingly (*C*_1_ to *C_k_*).
4:	**for** each remaining slice (*S_i_*) **do**
5:	Compute the overlapping of each 2D cell (*c*_*i, j*_) in current slice (*S_i_*) with the bottom-most slice of each of the currently identified cells (*C_j_*) (provided that the bottom-most slice of that cell is at position *i* − 1, i.e., both slices are directly touching; if they are not touching, the overlap is zero).
6:	If cell *c*_*i, j*_ overlaps similarly with several bottom-most slices (*c*_*i* − 1, *l*_, *c*_*i* − 1, *m*_) of several cells (*C*_*l*_, *C*_*m*_), cell *c*_*i, j*_ is divided into multiple parts. To consider two or more overlappings of similar size, the difference between them can not exceed a given threshold (δ_1_).
7:	For the remaining cells, assign each 2D cell (*c*_*i, j*_) of current slice (*S_i_*) to the 3D cell it overlaps the most, given that this overlapping is over a minimum threshold (δ_2_).
8:	Cells not satisfying the minimum overlapping threshold δ_2_ are assigned to a new 3D cell (*C*_*k* + 1_).
9:	**end for**
10:	**end for**

Algorithm 1 processes 3D connected components such as those represented in Figures 3 or 5. Note that these images have already been segmented in 2D and, for that reason, some of the slices are already divided (for example, Figures 3A and 5E).

***2.3.4.1. Correcting over-segmentation.*** Figures 3, 4 show an example of how 2D over-segmentation due to a wrong binarization can be corrected by the proposed algorithm. In Figure 4A, both 2D cells are assigned different labels (colors) (step 3 of Algorithm 1). Then, for each of the remaining slices, each cell is assigned the label (color) of the cell in the previous slice that it overlaps with the most (step 7 of Algorithm 1). It should be noted how the 2D over-segmentation present in Figure 4F is corrected in 3D by assigning both parts of the cell the same label (color) as their maximum overlapping corresponds to the same cell in the previous slice.

***2.3.4.2. Correcting under-segmentation.*** Figures 5, 6 show an example of how 2D under-segmentation can be corrected with the 3D reconstruction algorithm. Figure 5 represents the 2D segmentation of a cluster of cells. In this example, the 2D segmentation algorithm was able to correctly split two adjacent cells in slices Figures 5E,F, but not in slice 5G. The 3D reconstruction algorithm is able to correct this problem by comparing the segmentation in slice Figure 5G with that of the previous slice (step 6 of Algorithm 1). As the only identified cell in this slice overlaps in a similar way with two cells in the previous slice, it is divided into two cells and assigned corresponding labels (colors), as can be seen in Figure 6G.

#### 2.3.5. Post-processing 3D segmentations

The results obtained by using the approach described in Algorithm 1 are quite good in terms of correctly segmented cell nuclei. However, there are a small number of cases showing mis-segmentations associated with the particular images and/or the binarization process. This issues can be overcome in a number of different ways (which are outlined below).

***2.3.5.1. Centroid-Clustering under-segmentation correction.*** The first improvement deals with clusters of cells that could not be split at any of the slices (this is a different case to that previously shown in Figures 5, 6 where the under-segmentation only occurs in some slices and the information present in the remaining slices can be used to correctly segment the 3D cells). Figure 7 shows an example where it was not possible to split cells at any of the slices. In this image, we can see how the 2D segmentation algorithm is unable to divide the two cells present in slices Figures 7F,G. To correct this type of situation, we have followed an approach based on the analysis of the centroids of each cell at each slice. Our hypothesis is that, if there are significant differences in the position of the centroids, this means that there are two or more adjacent cells in the image. However, it is difficult to determine how many cells there are in these clusters, although it is safe to assume that the number is small since most of the cells have already been divided.

Algorithm 2 describes how the number of cells can be determined. This algorithm uses the k-means algorithm to create different sets of clusters (with different *k* values) and analyzes the intra-cluster distance for each *k* value. The number of cells in a cluster would be the number that minimizes the intra-cluster distance, given that every cluster has a minimum size (α parameter) and that the ratio between the sizes of every two pairs of clusters is not smaller than a given ratio (β parameter).

**Algorithm 2:** **Determining the number of adjacent cells based on centroid positions**.

1:	Initialize an empty list of centroids: *C*
2:	**for** every slice *S_i_* **do**
3:	Compute the centroid of the cell: *C_i_*
4:	**end for**
5:	Initialize an empty list of intra-cluster distances: *D*
6:	**while** *k* < *maxClusters* **do**
7:	Run k-means on *C* with *k* as the number of clusters and store the average intra-cluster
	distance, *D_k_*
8:	**if** size of any cluster is smaller than α **then** *D_k_* = *inf*
9:	**end if**
10:	**if** size ratio for any two clusters is smaller than β **then** *D_k_* = *inf*
11:	**end if**
12:	**end while**
13:	Number of cells equals *k* minimizing *D_k_* (*cells* = *argmin*(*D_k_*))

Now that a candidate number of cells has been selected, the cells must be split according to the clusters of centroids identified by Algorithm 2. Prior to this division, the algorithm tests whether the clusters found actually represent multiple cells by examining the slices at the borders of the identified clusters. Taking Figure 7 as an example, and assuming that Algorithm 2 identifies two clusters, it would compare slices Figures 7E,F for the first cluster, and slices Figures 7G,H for the second one. For each pair of slices, the algorithm computes the degree of cell overlapping. If the overlapping of any of the pairs of slices is below a given threshold (γ), the algorithm assumes that there are multiple cells and it can proceed to the actual division of the clusters into individual cells. For each two consecutive clusters of centroids, the centroid of the first slice of the first cluster and the centroid of the last slice of the second cluster are used as reference points. Pixels from each slice of both clusters are then assigned to the reference point that they are closest to. At the end of this process, there will be as many labeled cells as there are clusters, as can be seen in Figure 8.

***2.3.5.2. Filtering out small segmentations.*** The second improvement deals with the erasing of spurious small segmentations. There are a few cases in which an inaccurate 2D segmentation leads to small pieces of cells being identified as individual cells in a single slice (see Figure 9A). In these cases, there are two options: (1) add the small piece to its closest cell nucleus or (2) remove these spurious cell nuclei, as shown in Figure 9B. In our case, we have decided to follow the second approach, as most errors are typically produced when dye staining also fills portions of the closest part to the nucleus, causing an irregular shaped cell body to appear in a single slice. Due to this shape, these regions are sometimes labeled as different 2D structures in one or two slices.

### 2.4. Testing environment

The results reported in the present study have been obtained with the following computer configuration and programming language: PC, Intel Core i7-2600K 4 cores 3.4 Ghz CPU; Operating System, Ubuntu Linux 12.10; Programming Language, Matlab R2011b.

## 3. Results and discussion

The proposed algorithm was applied to a set of 3 image stacks (from layers 2, 3 and 6) of the somatosensory neocortex from 14-day-old rats. These images were selected as representative in terms of the density and distribution of neurons in these cortical layers (layer 2, high density; layers 3 and 6, intermediate density). They also constitute a good benchmark as the number of neurons in each stack of images is relatively high, ranging from approximately 196 to 243 neurons each.

Table 1 contains the parameter values used in this study. All of these parameters were chosen experimentally and in a conservative way. For example, the parameters used in the division of clusters of cells by analyzing the centroids were selected to ensure that cells which had already been correctly segmented are not over-segmented by the use of this improvement.

**Table 1:** **Parameter values of the proposed algorithm**.

**Parameter**	**Values**
Similar overlapping threshold δ_1_	0.2
Minimum overlapping threshold δ_2_	0.2
k-means iterations *maxClusters*	3
Minimum size of cluster α	3
Size ratio for clusters β	0.3
Maximum overlapping of clusters γ	0.65

All the parameters were chosen experimentally and in a conservative way. For example, the parameters used in the division of clusters of cells by analyzing the centroids were selected to ensure that cells which had already been correctly segmented are not over-segmented by the use of this improvement.

### 3.1. Comparison of performance with different 2D algorithms

To validate the accuracy of the proposed algorithm when used with different 2D segmentation techniques, the segmented images obtained have been reviewed by an expert in the field of neuroanatomy. The validation procedure involves the expert manually validating the proposed outcome of a reference algorithm (we selected the most promising one, which was the BinCS configuration). The resulting validated dataset was considered the benchmark against which the rest of the configurations could be validated in a semi-automated way (i.e., our “ground truth”).

Table 2 summarizes the results obtained in terms of correctly segmented nuclei and incorrect segmentations, according to the manual validation of the expert. In order to provide more insight into the behavior of the algorithm, we have reported the possible different types of error separately.

**Table 2:** **Manual validation results for the BinCS 2D algorithm**.

	**Layer 2 (%)**	**Layer 3 (%)**	**Layer 6 (%)**
Correct	90.82	91.34	94.17
Type-1 error: over-segmentation	4.35	3.94	4.93
Type-2 error: under-segmentation	3.86	4.33	0.00
Type-3 error: noise detected as cell	0.97	0.39	0.90
Type-4 error: undetected cell	0.00	0.00	0.00

Results of a manual validation carried out by an expert neuroanatomist. For each labeled 3D neuronal nucleus, the expert decides if it corresponds to a correctly segmented neuronal nucleus or if it constitutes one of the four different errors considered here.

For the remaining configurations, a semi-automatic validation approach was used. To conduct such a validation, the images obtained after the 2D segmentation are taken into consideration, for both the BinCS configuration (our reference algorithm) and each of the other configurations. For each of the four stacks, the 3D connected components are obtained for both the BinCS configuration and the algorithm that must be validated. With these 3D connected components, we compute clusters of 3D structures coming from both algorithms that highly overlap among them, given a threshold (*th*). If a cell detected by the BinCS algorithm has not been identified by the other algorithm, it is marked as an “undetected cell,” whereas a cell appearing only in the algorithm to be validated and not in the results of the BinCS algorithm is marked as “noise detected as cell”. We assessed the suitability of this approach using several threshold values (75, 85, 90, and 95%).

With the clusters of objects that overlap to a large extent which are obtained from both algorithms (reference algorithm and validating algorithm), an automatic labeling process is conducted following the rules shown in Algorithm 3. Image segmentation algorithms that use non-synthetic data require a manual validation by a domain expert. To improve on this limitation, we considered it extremely important to try to provide a semi-automatic procedure to re-use the expert's validation session to test the segmented structures from different algorithms, thereby requiring expert intervention in only a small number of cases.

**Algorithm 3:** **Automatic labeling of clusters of highly overlapping objects**.

1:	Let a set of cells identified by the Reference Algorithm (Ground Truth) be *A*_1_ and a set of cells detected by the Validating Algorithm be *A*_2_. The union of both sets of cells *A*_1_ and *A*_2_ is the cluster under consideration.
2:	**if** size(*A*_1_)==1 and size(*A*_2_)==1 **then** *A*_2_[1].label=*A*_1_[1].label
3:	**else if** size(*A*_1_)==1 and size(*A*_2_)=2 **then**
4:	**if** *A*_1_[1].label==“True Positive” **then**
5:	*A*_2_[1].label=“Oversegmented”
6:	*A*_2_[2].label=“Oversegmented”
7:	**else if** *A*_1_[1].label==“Undersegmented” **then**
8:	*A*_2_[1].label=“True Positive”
9:	*A*_2_[2].label=“True Positive”
10:	**else**
11:	*A*_2_[1].label=“Manual Validation”
12:	*A*_2_[2].label=“Manual Validation”
13:	**end if**
14:	**else if** size(*A*_1_)==2 and size(*A*_2_)=1 **then**
15:	**if** *A*_1_[1].label==“True Positive” and *A*_1_[2].label==“True Positive” **then**
16:	*A*_2_[1].label=“Undersegmented”
17:	**else if** *A*_1_[1].label==“Oversegmented” or *A*_1_[2].label==“Oversegmented” **then**
18:	*A*_2_[1].label=“True Positive”
19:	**else**
20:	*A*_2_[1].label=“Manual Validation”
21:	**end if**
22:	**else**
23:	**for** *i* = 1; *i* <= *size*(*A*_2_); *i* ++ **do**
24:	*A*_2_[*i*].label=“Manual Validation”
25:	**end for**
26:	**end if**

Depending on the overlapping threshold value used, the number of cells that must still be manually validated will vary accordingly, ranging from 10–15% for *th* = 75% to 40–45% for *th* = 95%. If the validation values with different thresholds are similar, it is preferable to use a threshold of 75% since it is significantly quicker than using a threshold of 95%.

Tables 3–5 contain the validation values obtained semi-automatically for each 2D algorithm, stack of images and threshold value. From these data it can be seen that there are not big differences between the validation values obtained with the different thresholds (normally around 1%, and no more than 2.5% in the worst case). Furthermore, the better the 2D segmentation is, the smaller the fluctuation in the validation values for different threshold values (for example, in the case of the BinWS configuration there are no differences at all).

**Table 3:** **Semi-automatic validation results for the BinWS 2D algorithm**.

	**th = 75%**	**th = 85%**	**th = 90%**	**th = 95%**
**LAYER 2**				
Correct	92.54	92.54	92.54	92.54
Type-1 error: over-segmentation	3.98	3.98	3.98	3.98
Type-2 error: under-segmentation	3.48	3.48	3.48	3.48
Type-3 error: noise detected as cell	0.00	0.00	0.00	0.00
Type-4 error: undetected cell	0.00	0.00	0.00	0.00
**LAYER 3**				
Correct	89.11	89.11	89.11	89.11
Type-1 error: over-segmentation	3.50	3.50	3.50	3.50
Type-2 error: under-segmentation	7.00	7.00	7.00	7.00
Type-3 error: noise detected as cell	0.39	0.39	0.39	0.39
Type-4 error: undetected cell	0.00	0.00	0.00	0.00
**LAYER 6**				
Correct	95.07	95.07	95.07	95.07
Type-1 error: over-segmentation	4.04	4.04	4.04	4.04
Type-2 error: under-segmentation	0.90	0.90	0.90	0.90
Type-3 error: noise detected as cell	0.00	0.00	0.00	0.00
Type-4 error: undetected cell	0.00	0.00	0.00	0.00

Results of a semi-automatic validation for the BinWS 2D algorithm and several overlapping threshold values. The results obtained are very similar to those of the BinCS 2D algorithm (reported in Table 2). Furthermore, a similar performance was achieved at different threshold values and thus smaller values can be safely used, which minimizes manual intervention by the expert.

**Table 4:** **Semi-automatic validation results for the LSCS 2D algorithm**.

	**th** = **75%**	**th** = **85%**	**th** = **90%**	**th** = **95%**
**LAYER 2**				
Correct	79.49	79.49	79.91	80.34
Type-1 error: over-segmentation	17.52	15.81	15.38	14.96
Type-2 error: under-segmentation	2.99	4.70	4.70	4.70
Type-3 error: noise detected as cell	0.00	0.00	0.00	0.00
Type-4 error: undetected cell	0.00	0.00	0.00	0.00
**LAYER 3**				
Correct	78.95	79.71	78.91	78.55
Type-1 error: over-segmentation	13.33	14.13	14.91	14.91
Type-2 error: under-segmentation	7.02	5.43	5.45	5.82
Type-3 error: noise detected as cell	0.70	0.72	0.73	0.73
Type-4 error: undetected cell	0.00	0.00	0.00	0.00
**LAYER 6**				
Correct	80.08	79.17	80.00	80.42
Type-1 error: over-segmentation	19.50	19.17	17.92	17.92
Type-2 error: under-segmentation	0.41	1.67	2.08	1.67
Type-3 error: noise detected as cell	0.00	0.00	0.00	0.00
Type-4 error: undetected cell	0.00	0.00	0.00	0.00

Results of a semi-automatic validation for the LSCS 2D algorithm and several overlapping threshold values. It can be seen that, similar to observations with the BinWS algorithm, a similar performance was achieved at different threshold values and thus smaller values can be safely used, which minimizes manual intervention by the expert.

**Table 5:** **Semi-automatic validation results for the LSWS 2D algorithm**.

	**th** = **75%**	**th** = **85%**	**th** = **90%**	**th** = **95%**
**LAYER 2**				
Correct	79.57	79.13	81.50	80.62
Type-1 error: over-segmentation	13.48	13.04	12.33	13.22
Type-2 error: under-segmentation	6.09	6.96	5.29	5.29
Type-3 error: noise detected as cell	0.87	0.87	0.88	0.88
Type-4 error: undetected cell	0.00	0.00	0.00	0.00
**LAYER 3**				
Correct	78.83	79.70	79.09	78.41
Type-1 error: over-segmentation	12.41	13.16	12.55	12.88
Type-2 error: under-segmentation	7.66	6.02	7.22	7.20
Type-3 error: noise detected as cell	1.09	1.13	1.14	1.52
Type-4 error: undetected cell	0.00	0.00	0.00	0.00
**LAYER 6**				
Correct	80.59	79.74	81.03	82.76
Type-1 error: over-segmentation	16.46	17.24	15.52	13.36
Type-2 error: under-segmentation	2.53	2.59	3.02	3.02
Type-3 error: noise detected as cell	0.42	0.43	0.43	0.86
Type-4 error: undetected cell	0.00	0.00	0.00	0.00

Results of a semi-automatic validation for the LSWS 2D algorithm and several overlapping threshold values. It can be seen that, similar to observations with the BinWS and LSCS algorithms, a similar performance was achieved at different threshold values and thus smaller values can be safely used, which minimizes manual intervention by the expert. Furthermore, the results are very similar to those of the LSCS algorithm (reported in Table 4), which confirms that the key element of the 2D segmentation seems to be the binarization step, regardless of the algorithm used for the division of clusters.

From these tables, we can also see that the critical factor in the 2D validation (at least for the configurations considered) seems to be the algorithm used in the initial segmentation phase; both configurations using the Two-step Binarization obtain significantly better results than those using Level Set Methods, regardless of whether the cluster separation algorithm used was clump splitting or Watershed. Furthermore, the results of both configurations using the Two-step Binarization are very similar (with only small differences of 1-2 more cells detected by one algorithm or the other). Hence, we can conclude from this comparison that an effective 2D segmentation is critical for obtaining an accurate 3D reconstruction of cells.

### 3.2. Comparison with watershed in 3D

The proposed 3D reconstruction algorithm has been compared with the direct application of the Watershed algorithm in 3D. To conduct this comparison, we took the results of the BinCS configuration depicted in Table 2 as a reference — since there were small differences between two of the 2D algorithms compared in the previous section.

Table 2 showed that the proposed algorithm obtains accurate results in all the stacks of images coming from different layers. The accuracy is especially good for layer 6, which would be as expected since this is the layer in which the 2D segmentation also worked the best. Moreover, the error values were moderate for all error types. In our opinion, over-segmentation seems to be the most problematic and difficult error to tackle, as the over-segmentation of a single cell in a single slice constitutes an over-segmentation error. The proposed algorithm is able to correct some of these errors (see Figure 4) but not all of them. Specific corrections for this type of error seem to be necessary. However, these errors are normally easy to detect as the over-segmented cells usually form small structures that can be removed in a post-processing step before obtaining statistics on the segmented images.

Regarding the Watershed algorithm, we have used the Matlab version, which is implemented according to work by Meyer (1994). The results obtained by the Watershed algorithm are reported in Table 6. From these results, it can be seen that the Watershed transform “as is” is not able to deal with this type of problem. There is a clear over-segmentation issue that must be specifically addressed.

**Table 6:** **Semi-automatic validation results for the 3D Watershed algorithm (Matlab implementation)**.

	**Layer 2 (%)**	**Layer 3 (%)**	**Layer 6 (%)**
Correct	46.48	44.09	43.13
Type-1 error: over-segmentation	49.65	54.33	56.56
Type-2 error: under-segmentation	3.87	1.31	0.31
Type-3 error: noise detected as cell	0.00	0.26	0.00
Type-4 error: undetected cell	0.00	0.00	0.00

We used Matlab's implementation, which is programmed according to work by Meyer (1994). It can be observed that Watershed, if used without any further refinement, is not suitable for this problem, as it suffers from a large over-segmentation problem in all the stacks of images considered.

A possible solution to improve the results obtained using the Watershed algorithm in this kind of image is to try to fuse adjacent cells by modifying the distance matrix. Amira's implementation of this algorithm (Stalling et al., 2005) [based on work by Yoo et al. (2002) available in the Insight Toolkit library] includes a parameter called “Minimal Depth” that conducts such a transformation. Table 7 contains the results obtained with the Watershed 3D implementation included in Amira. The “Minimal Depth” parameter takes values ranging from 2.2 to 2.8, approximately, depending on the image. It can be seen that the dramatic over-segmentation of Matlab's implementation is no longer there and that the overall number of correctly segmented cells has increased. However, now the percentage of under-segmented cells has significantly increased. Decreasing the value of the “Minimal Depth” parameter helps to decrease the number of under-segmented cells but, on the other hand, it makes the number of over-segmented cells increase rapidly. It is thus difficult, if not impossible, to find a good compromise value for the “Minimal Depth” parameter that minimizes both types of error simultaneously, probably due to the irregular shape of the cell nuclei in the input images.

**Table 7:** **Semi-automatic Validation Results for the 3D Watershed algorithm (Amira's implementation)**.

	**Layer 2 (%)**	**Layer 3 (%)**	**Layer 6 (%)**
Correct	58.18	70.16	80.18
Type-1 error: over-segmentation	0.45	1.16	1.76
Type-2 error: under-segmentation	41.36	28.68	18.06
Type-3 error: noise detected as cell	0.00	0.00	0.00
Type-4 error: undetected cell	0.00	0.00	0.00

We used Amira's implementation of this algorithm Stalling et al. (2005) (based on work by Yoo et al. (2002) available in the Insight Toolkit library). This implementation of the algorithm includes a parameter called “Minimal Depth” that attempts to fuse adjacent cells by modifying the distance matrix and thus correct the over-segmentation problem of this algorithm. It can be seen that this implementation improves the results obtained by the standard Watershed algorithm, but provides much less of an improvement than that seen with the algorithm proposed in the present paper.

It should be noted that, in most of the cases, all the alternative configurations for 3D reconstruction from 2D segmentations that were considered obtained better results than either of the two Watershed implementations tested. This gives an idea of the difficulty of working directly on the 3D problem and how a 3D reconstruction algorithm can help to tackle this problem.

Finally, there are other authors that used a similar problem-specific, tailor-made approach to reduce the number of local maxima in the input images. For example, in the work by Oberlaender et al. (2009), though the described algorithm does not solve exactly the same problem discussed in this paper (it only counts neurons, and does not perform any actual segmentation of the volumes), the distance matrix is modified in such a way that local maxima separated by less than a minimum value are considered to be the same. With this modification, the results are better than those obtained with the basic Watershed algorithm. However, there are further post-processing steps such as, for example, size-based clustering, so the actual influence of this modification is not clear. In any case, in a simplified version of the problem (involving counting alone) the results obtained by this algorithm are quantitatively less efficient than the results reported in the present work. Moreover, Oberlaender's counting benefits from compensated errors (between over- and under-segmented cases). In our work, which involves not only counting but also the correct identification of the cells, we are able to correctly identify between 91% and 94% of the cells.

## 4. Conclusions

In this paper, we have presented an algorithm to reconstruct 3D cells from 2D segmented cells. This approach can be very useful as (1) it makes use of efforts already put into developing accurate 2D segmentation methods; and (2) it is independent of the 2D segmentation algorithm or the images being studied, which allows the algorithm to be used on different problems by simply selecting the most appropriate 2D segmentation algorithm. Moreover, the proposed method has been tested in a real scenario—the segmentation of 684 neuronal nuclei in the rat neocortex.

We have tested the algorithm with different 2D segmentation algorithms and the quality of the results obtained depends on the quality of 2D segmentations: the better the 2D segmentation is, the better the 3D reconstruction is. In our experiments, similar results were obtained using two 2D algorithms, which both use the same binarization algorithm. Compared to the traditional 3D Watershed algorithm, the proposed algorithm obtained significantly better results in terms of correctly identified cell nuclei. Nonetheless, even though the over-segmentation error has been kept to a moderate value, future work should focus on dealing with this specific problem.

### Conflict of interest statement

The authors declare that the research was conducted in the absence of any commercial or financial relationships that could be construed as a potential conflict of interest.
